# Modified-Release and Conventional Glucocorticoids and Diurnal Androgen Excretion in Congenital Adrenal Hyperplasia

**DOI:** 10.1210/jc.2016-2855

**Published:** 2016-11-15

**Authors:** Christopher M. Jones, Ashwini Mallappa, Nicole Reisch, Nikolaos Nikolaou, Nils Krone, Beverly A. Hughes, Donna M. O’Neil, Martin J. Whitaker, Jeremy W. Tomlinson, Karl-Heinz Storbeck, Deborah P. Merke, Richard J. Ross, Wiebke Arlt

**Affiliations:** 1 Institute of Metabolism and Systems Research, University of Birmingham, Birmingham, B15 2TT, United Kingdom; 2 National Institutes of Health Clinical Center and the Eunice Kennedy Shriver National Institute of Child Health and Human Development, Bethesda, Maryland 20892; 3 Medizinische Klinik und Poliklinik IV, Ludwig-Maximilians-Universität München, 80366 Munich, Germany; 4 Oxford Centre for Diabetes, Endocrinology & Metabolism, University of Oxford, Oxford, OX3 7LE, United Kingdom; 5 Academic Unit of Endocrinology, Department of Human Metabolism, University of Sheffield, Sheffield S10 2RX, United Kingdom; 6 Diurnal Ltd., Cardiff, CF14 4UJ, United Kingdom; 7 Department of Biochemistry, University of Stellenbosch, Stellenbosch 7600, South Africa; 8 Centre for Endocrinology, Diabetes and Metabolism, Birmingham Health Partners, Birmingham, B15 2TH, United Kingdom

## Abstract

**Context:**

The classic androgen synthesis pathway proceeds via dehydroepiandrosterone, androstenedione, and testosterone to 5*α*-dihydrotestosterone. However, 5*α*-dihydrotestosterone synthesis can also be achieved by an alternative pathway originating from 17*α*-hydroxyprogesterone (17OHP), which accumulates in congenital adrenal hyperplasia (CAH). Similarly, recent work has highlighted androstenedione-derived 11-oxygenated 19-carbon steroids as active androgens, and in CAH, androstenedione is generated directly from 17OHP. The exact contribution of alternative pathway activity to androgen excess in CAH and its response to glucocorticoid (GC) therapy is unknown.

**Objective:**

We sought to quantify classic and alternative pathway-mediated androgen synthesis in CAH, their diurnal variation, and their response to conventional GC therapy and modified-release hydrocortisone.

**Methods:**

We used urinary steroid metabolome profiling by gas chromatography–mass spectrometry for 24-hour steroid excretion analysis, studying the impact of conventional GCs (hydrocortisone, prednisolone, and dexamethasone) in 55 adults with CAH and 60 controls. We studied diurnal variation in steroid excretion by comparing 8-hourly collections (23:00–7:00, 7:00–15:00, and 15:00–23:00) in 16 patients with CAH taking conventional GCs and during 6 months of treatment with modified-release hydrocortisone, Chronocort.

**Results:**

Patients with CAH taking conventional GCs showed low excretion of classic pathway androgen metabolites but excess excretion of the alternative pathway signature metabolites 3*α*,5*α*-17-hydroxypregnanolone and 11*β*-hydroxyandrosterone. Chronocort reduced 17OHP and alternative pathway metabolite excretion to near-normal levels more consistently than other GC preparations.

**Conclusions:**

Alternative pathway-mediated androgen synthesis significantly contributes to androgen excess in CAH. Chronocort therapy appears superior to conventional GC therapy in controlling androgen synthesis via alternative pathways through attenuation of their major substrate, 17OHP.

## Teaser

We studied diurnal urinary steroid excretion in glucocorticoid-treated patients with congenital adrenal hyperplasia and found increased alternative pathway androgen synthesis that was ameliorated by modified-release hydrocortisone.

Disruption of glucocorticoid (GC) synthesis is the defining feature of all variants of congenital adrenal hyperplasia (CAH), including its most prevalent cause, 21-hydroxylase deficiency (21OHD) (1). This enzymatic block results in GC deficiency, with the consequent loss of negative feedback to the pituitary gland and hypothalamus, driving both adrenocorticotropic hormone (ACTH)-mediated adrenal androgen excess and adrenal hyperplasia. Mineralocorticoid deficiency may also be seen in 21OHD but to a variable degree dependent on mutation severity (2).

**Figure 1.**
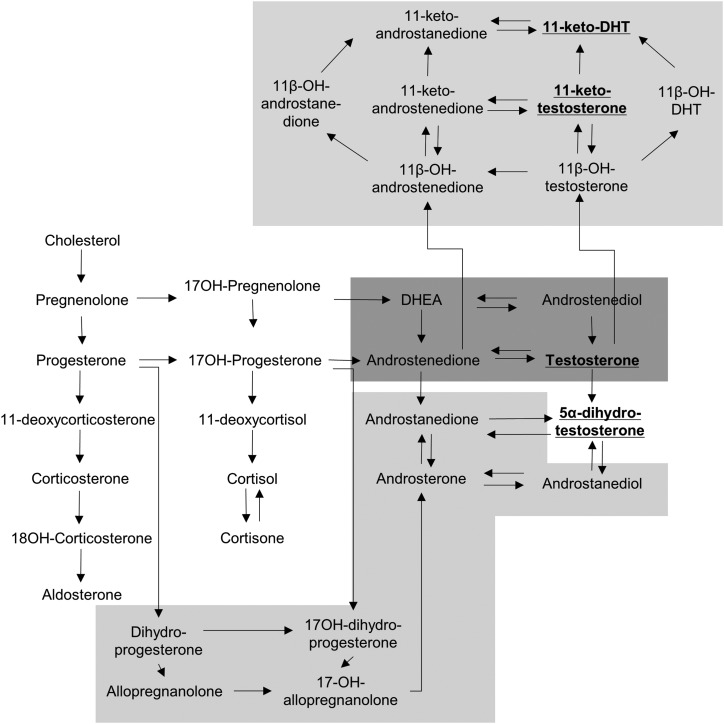
Schematic overview of steroidogenesis. The graph depicts steroidogenesis, including the classic androgen synthesis pathway (shaded in dark gray) and the 2 alternative androgen synthesis pathways (shaded in light gray; top, 11-oxygenated 19-carbon steroids; bottom, alternative pathway to DHT). 3*α*,5*α*-17HP is labeled by its alternative full name, 17-OH-allopregnanolone.

The classic pathway of androgen synthesis proceeds through dehydroepiandrosterone (DHEA), androstenedione, and testosterone to the most potent activator of the androgen receptor, 5*α*-dihydrotestosterone (DHT). The substrate of 21-hydroxylase, 17*α*-hydroxyprogesterone (17OHP), accumulates in CAH due to 21OHD, resulting in enhanced conversion to androstenedione and active androgens. However, 17OHP is also a substrate for an alternative pathway to androgen biosynthesis, which generates DHT without the need for DHEA, androstenedione, or testosterone as intermediates (3, 4). In this pathway, 17OHP is converted by consecutive 5*α*-reductase and 3*α*-hydroxysteroid dehydrogenase activity to 3*α*,5*α*-17-hydroxypregnanolone (3*α*,5*α*-17HP) and then downstream to DHT (Fig. 1) (5). Accumulation of the alternative pathway intermediate 3*α*,5*α*-17HP has been demonstrated in untreated patients with CAH due to 21OHD (6), but its relative contribution to excess androgen synthesis has not yet been investigated. Furthermore, recent work has highlighted the role of another androgen biosynthesis pathway that converts androstenedione in several steps to 11-keto-testosterone and 11-keto-dihydrotestosterone (Fig. 1), steroids that have been shown to act as potent androgen receptor agonists (7–11).

Conventional management strategies for CAH include the use of both immediate-release hydrocortisone and longer-acting synthetic GC preparations, sometimes prescribed in a reverse circadian pattern (1). These preparations fail to mimic the normal diurnal profile of cortisol secretion and therefore do not prevent the early morning surge of ACTH that is the major driver of adrenal-mediated androgen excess in CAH. As a consequence, the current management of patients with CAH is complicated by the need to strike a balance between sufficient control of endogenous androgen excess and potential excess exposure to exogenous GCs (12). A modified-release and delayed-release GC preparation, Chronocort, has recently been developed and shown to approximate the physiological diurnal rhythm of cortisol release due to delayed release, with peak levels during the early morning hours after intake at bedtime (13, 14). The relative impact of both conventional GC preparations and Chronocort on androgen synthesis by classic and alternative pathways is not known.

In this study, we sought to quantify the diurnal contribution of alternative pathway androgen synthesis to androgen excess in CAH by assessing the excreted urinary steroid metabolome of patients with 21OHD. We investigated patients receiving conventional GC therapy and patients treated with the modified-release hydrocortisone preparation Chronocort in comparison with healthy controls with intact diurnal secretion of cortisol.

## Patients and Methods

### Patients

Alternative pathway androgen synthesis in patients with CAH managed with conventional GC therapy was quantified by analysis of 24-hour urinary steroid metabolite excretion in 55 adult patients with 21OHD, recruited from 2 specialist centers, Birmingham and Munich, and 60 sex- and age-matched controls, recruited from Birmingham. In all participating patients, the diagnosis of 21OHD had previously been confirmed following genetic testing as part of their routine clinical care. Control participants were healthy individuals without chronic disease aged 18 to 80 years. None were taking oral contraceptives, hormone replacement therapy other than corticosteroid replacement, or other medications known to alter steroid hormone synthesis and/or metabolism at the time of urine collection.

A summary of patient and control characteristics is provided in Table 1. Most of the 21OHD group was managed with prednisolone (49%, n = 27; median daily dose, 7.5 mg; range, 5–15 mg) and the remainder with either hydrocortisone (24%, n = 13; median daily dose, 30 mg; range, 20–37.5 mg) or dexamethasone (27%, n = 15; median daily dose, 0.5 mg; range, 0.25–1.00 mg). All patients with salt-wasting CAH and some with simple-virilizing CAH received additional mineralocorticoid replacement; daily fludrocortisone doses ranged between 100 and 300 μg.

**Table 1.** **Demographic Data for Patients With CAH Managed With Conventional GC Treatment or Chronocort and for Healthy Matched Control Participants**

Characteristic	**CAH Patients Taking Conventional GC Therapy (n = 55)**	**Control Cohort (n = 60)**	**Chronocort-Treated Patients With CAH (n = 16)**	**Control Cohort (n = 12)**
Male/female, n/n	28/27	32/28	8/8	12/0
Age, median (range), y	31 (19–49)	26 (20–48)	24 (18–60)	28.5 (22–60)
CAH phenotype, n (%)				
Salt-wasting	41 (74.5)	NA	12 (75)	NA
Simple virilizing	14 (25.5)	NA	4 (25)	NA
GC preparation, n (%)				
Hydrocortisone	13 (24)	NA	3 (19)	NA
Prednisolone	27 (49)	NA	8 (50*)*^a^	NA
Dexamethasone	15 (27)	NA	5 (31)	NA

For Chronocort-treated patients, their conventional GC medication prior to commencing Chronocort therapy is shown.

Abbreviation: NA, not applicable.

^*a*^ One patient received a combined hydrocortisone and prednisolone preparation prior to commencing Chronocort therapy and has been included in the prednisolone group.

The impact of the modified-release hydrocortisone preparation Chronocort on alternative pathway synthesis was assessed in a subgroup consisting of 16 patients with 21OHD. All were enrolled in an open-label phase 2 study at the National Institutes of Health Clinical Centre (clinicaltrials.gov, NCT01735617) (14). Patients were maintained on twice-daily Chronocort therapy for 6 months with dose adjustment used based on clinical symptoms and serum biochemistry. Median daily Chronocort dose at 6 months was 27.5 (range, 15–40) mg. Urinary steroid metabolite excretion was measured at baseline, at day 4 of Chronocort therapy, and after 6 months of treatment. Three 8-hour urine samples were collected within each of these three 24-hour periods and were timed to reflect either night (23:00–07:00), morning (07:00–15:00), or evening (15:00–23:00) periods. Steroid excretion in the 8-hourly urine collection was compared with that of 12 healthy control participants (median age, 32.9 years) who also provided three 8-hour urine collections with similar timing to reflect night, morning, or evening periods. All participants provided informed written consent. Ethical approval for the collection of baseline data was provided by the South Birmingham Research Ethics Committee for healthy controls and by West Midlands Multicentre Research Ethics Committee and the University Hospital Ethics Committee Munich for conventionally managed patients with CAH. Phase 2 study approval for the Chronocort-treated patients with CAH was provided by the Eunice Kennedy Shriver National Institute of Child Health and Human Development Institutional Review Board at the National Institutes of Health.

### Urinary steroid hormone analysis

Analysis of urinary excretion of steroid hormone metabolites was undertaken by quantitative gas chromatography–mass spectrometry in selected ion-monitoring analysis mode as described previously (7). Supplemental Table 1 summarizes the steroid metabolites relevant to this study.

The 21-hydroxylase enzyme, CYP21A2, catalyzes the conversion of 17OHP to the cortisol precursor 11-deoxycortisol. The metabolic impact of 21OHD was thus assessed through analysis of tetrahydro-11-deoxycortisol, the metabolite of the CYP21A2 product 11-deoxycortisol, and the 17OHP metabolites 17HP and pregnanetriol, as well as pregnanetriolone (PTONE). PTONE is the metabolite of 21-deoxycortisol, which is generated from 17OHP by CYP11B1 and only produced in appreciable amounts in the absence of 21-hydroxylase activity (*i.e.*, in 21OHD).

Classic androgen pathway activity was measured by quantification of the major androgen metabolites androsterone (An) and etiocholanolone (Et). Activation of the alternative pathway to DHT was assessed through quantification of its signature metabolite, 3*α*,5*α*-17HP. Although androstenedione and testosterone both feed into An and Et, the most potent androgen, DHT, is only represented in the 5*α*-reduced androgen metabolite An. Thus, the Et pool is only enhanced by classic androgen pathway activity, whereas the An pool increases with DHT synthesis via both the classic and alternative pathways. We therefore used the ratio 3*α*5*α*-17HP/An as an estimate of the proportional contribution of the alternative pathway to androgen synthesis.

19-Carbon androgens oxygenated at position C-11 have been shown to be produced by the adrenal glands, and 11-keto-testosterone and 11-keto-DHT have been shown to activate the androgen receptor (7, 10). Therefore, we measured the concentration of the major metabolite of urinary 11-oxy-C_19_ steroid metabolites, 11*β*-hydroxyandrosterone (11*β*-OH-An).

### Statistical analysis

Data are presented as median and interquartile range unless otherwise stated. Analyses were undertaken using the nonparametric Mann-Whitney and Kruskal-Wallis with post hoc Dunn tests for unpaired analyses. Paired data were analyzed using the nonparametric Wilcoxon test with Bonferroni correction applied for repeated analyses. Statistical analyses were undertaken using SPSS Statistics 21 (SPSS, Inc., Chicago, IL) and *P* values <0.05 were considered statistically significant. All *P* values were 2-sided.

## Results

### 24-Hour steroid metabolite excretion in patients with CAH receiving conventional GC therapy

**Figure 2.**
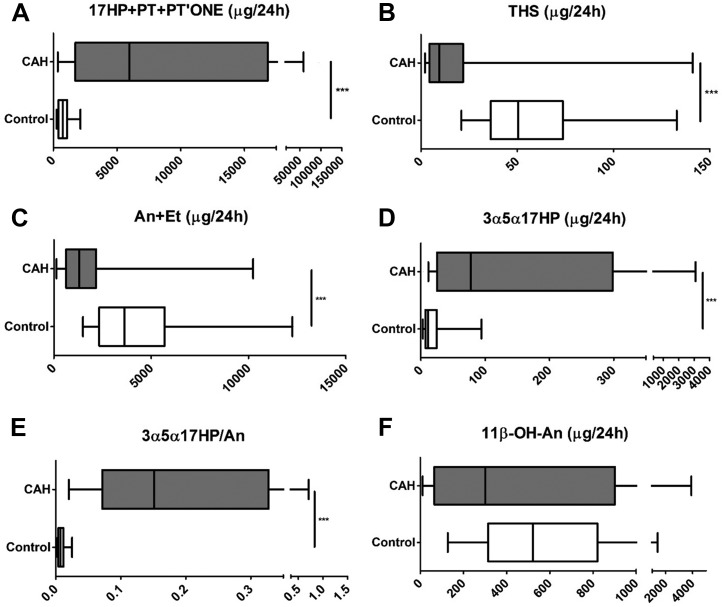
The 24-hour urinary steroid excretion in 55 patients with CAH and 60 healthy sex- and age-matched controls. For explanation of steroid metabolite abbreviations, see Table 1. (A) 17OHP metabolites. (B) 11-deoxycortisol metabolite. (C) Sum of active androgen metabolites. (D) Signature alternative pathway metabolite. (E) Ratio of alternative pathway/total 5*α*-reduced androgen metabolites. (F) Major 11-oxygenated androgen pathway metabolite. Data are shown as µg/24 hours and presented as box-and-whisker plots to represent median, interquartile range (box), and 5th and 95th percentiles (whiskers). Urinary excretion of 3*α*5*α*-17HP available for 38 of the total CAH cohort. Analyses were undertaken using the Mann-Whitney test. ***P ≤ 0.001 for CAH vs controls. PT, pregnanetriol; THS, tetrahydro-11-deoxycortisol.

As expected, the excretion of the 17OHP metabolites 17HP and pregnanetriol and the 21-deoxycortisol metabolite PTONE were significantly increased in CAH (*P* < 0.001), indicative of impaired 21-hydroxylase activity. Conversely, the product of 21-hydroxylase activity, the 11-deoxycortisol metabolite tetrahydro-11-deoxycortisol, was significantly lower (*P* < 0.001) in patients with CAH than in control participants [Figs. 2(A) and 2(B)].

The urinary excretion of the sum of the major androgen metabolites An and Et was significantly lower in patients with CAH managed with conventional GC therapy than in sex- and age-matched control participants [Fig. 2(C); *P* < 0.001]. Conversely, the signature metabolite of the alternative pathway to DHT synthesis, 3*α*5*α*-17HP, was significantly increased in patients with CAH [Fig. 2(D); *P* < 0.001]. The ratio of 3*α*,5*α*-17HP to An was calculated to quantify the contribution of the alternative pathway to total synthesis of 5*α*-reduced androgens, including DHT. This ratio was significantly increased in patients with CAH, whereas alternative pathway activity in the controls was negligible [*P* < 0.001; Fig. 2(E)]. The excretion of the major metabolite of 11-oxygenated 19-carbon steroids, 11*β*-OH-An, appeared similar to that in controls, with broad interindividual variability in excretion amounts [Fig. 2(F)]. The pattern of changes remained similar when carrying out sex-specific subgroup analyses (Supplemental Fig. 1), which also revealed higher excretion of the metabolites of 17OHP [Supplemental Fig. 1(A)] and classic and alternative pathway metabolites [Supplemental Fig. 1(C)–1(F)] in male controls compared with female controls, whereas no significant difference was observed between male and female patients with CAH.

### Diurnal variation in steroid excretion in patients with CAH receiving conventional GC therapy

**Figure 3.**
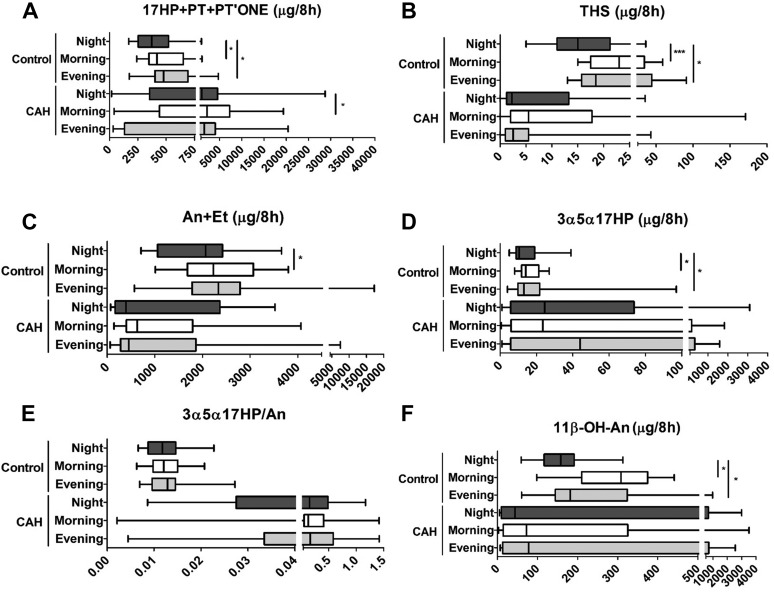
Eight-hourly diurnal urinary steroid metabolite excretion in 16 patients with CAH due to 21OHD and 12 healthy controls. (A) 17OHP metabolites. (B) 11-deoxycortisol metabolite. (C) Sum of active androgen metabolites. (D) Signature alternative pathway metabolite. (E) Ratio of alternative pathway/total 5*α*-reduced androgen metabolites. (F) Major 11-oxygenated androgen pathway metabolite. Data are shown for night (23:00–07:00; dark gray), morning (07:00–15:00; white), and evening (15:00–23:00; light gray) time periods. Excretion of the major androgen metabolites An + Et is shown for male patients with CAH (n = 8) and matched healthy controls (n = 12). Box-and-whisker plots represent median, interquartile range (box), and 5th and 95th percentiles (whiskers). Comparisons were drawn within CAH and control groups with analyses undertaken using the Friedman test, which was applied to the patients with CAH and the control participants separately. *P ≤ 0.05 for comparison of steroid excretion during different 8-hour periods. ***P ≤ 0.001. PT, pregnanetriol; THS, tetrahydro-11-deoxycortisol.

Diurnal excretion analysis in urines collected in 8-hour intervals reflecting night (23:00–07:00), morning (7:00–15:00), and evening (15:00–23:00) showed a similar picture to the 24-hour urine analysis when comparing patients with CAH (n = 16; 4 of whom were managed with hydrocortisone, 7 with prednisolone, and 5 with dexamethasone) to control participants (n = 12), with lower classic pathway but higher excretion of the signature metabolite of the alternative pathway to DHT in patients with CAH (Fig. 3).

Healthy control participants showed significant diurnal variability of the metabolites of 17OHP and the classic and alternative androgen pathways (Fig. 3), with lowest excretion during nighttime. By contrast, this diurnal excretion pattern was lost in patients with CAH receiving conventional GC therapy.

### Differential impact of conventional GC preparations on steroid excretion

To assess the effect of distinct conventional GC therapies on androgen synthesis, we compared urinary steroid metabolite excretion in patients with CAH managed with hydrocortisone (n = 13), prednisolone (n = 27), and dexamethasone (n = 15); all had been on stable treatment for at least 6 months. This revealed that hydrocortisone-treated patients with CAH had significantly higher excretion of 17OHP metabolites, the sum of the androgen metabolites An + Et and also the major adrenal androgen metabolite 11*β*-OH-An, in comparison with dexamethasone-treated patients, with prednisolone-treated patients in an intermediate position (Supplemental Fig. 2). Similarly, hydrocortisone therapy appeared associated with the highest excretion of the alternative pathway metabolite 3*α*,5*α*-17HP, but this difference was not statistically significant due to high interindividual variability.

### Diurnal steroid excretion during modified-release hydrocortisone treatment

We assessed urinary steroid excretion in 16 patients with CAH at baseline (*i.e.*, taking conventional GC treatment) and during treatment with modified-release hydrocortisone, with diurnal urine collections in 8-hourly intervals. This was carried out on 3 occasions: at baseline when still receiving conventional GC therapy, shortly after initiation of Chronocort treatment, day 4, and after 6 months of continuous treatment with Chronocort.

**Figure 4.**
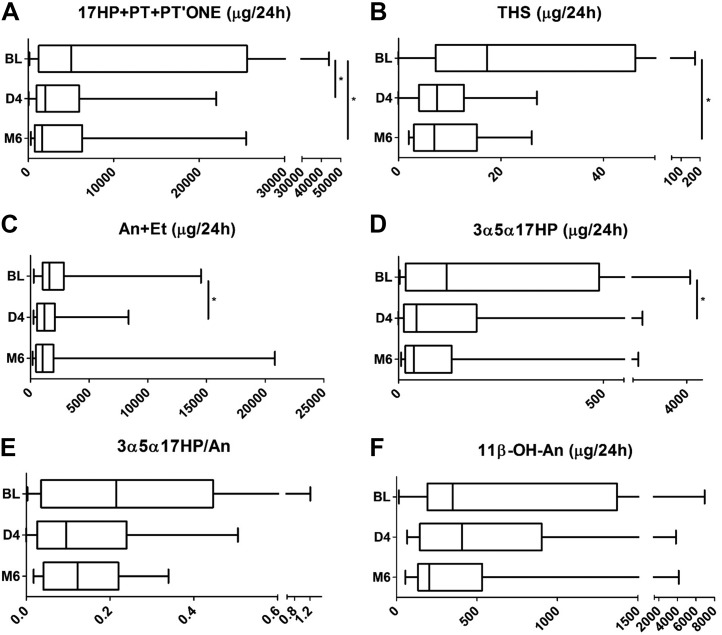
Effect of Chronocort treatment on 24-hour urinary steroid metabolite excretion in patients with CAH due to 21OHD. (A) 17OHP metabolites. (B) 11-deoxycortisol metabolite. (C) Sum of active androgen metabolites. (D) Signature alternative pathway metabolite. (E) Ratio of alternative pathway/total 5*α*-reduced androgen metabolites. (F) Major 11-oxygenated androgen pathway metabolite. Results are shown for patients with CAH at baseline taking conventional GC therapy prior to commencing Chronocort (BL; n = 16), at day 4 of Chronocort treatment (D4; n = 16), and after 6 months of Chronocort treatment (M6; n = 15). Box-and-whisker plots represent median, interquartile range (box), and 5th and 95th percentiles (whiskers). Analyses were undertaken using repeated Wilcoxon tests with Bonferroni correction to compare between matched patients with CAH. *P ≤ 0.05. PT, pregnanetriol; THS, tetrahydro-11-deoxycortisol.

**Figure 5.**
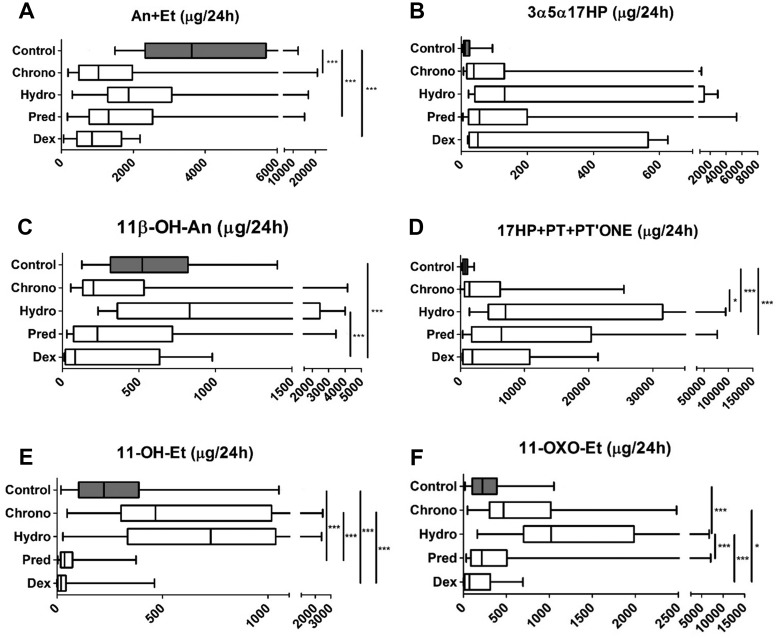
Urinary steroid excretion in 60 healthy controls and patients with CAH treated with Chronocort (n = 16), conventional immediate-release hydrocortisone (n = 13), prednisolone (n = 27), or dexamethasone (n = 15). (A) Sum of active androgen metabolites. (B) Signature alternative pathway metabolite. (C) 11*β*-hydroxyandrosterone, the major metabolite of the 11-oxygenated androgen pathway. (D) Sum of 17OHP metabolites. (E) Glucocorticoid metabolite 11-hydroxy-etiocholanolone. (F) Glucocorticoid metabolite 11-oxo-etiocholanolone. Urinary excretion of 3*α*5*α*-17HP available for 54 of the total CAH cohort; 16 on Chronocort, 11 on conventional hydrocortisone, 21 on prednisolone, and 6 on dexamethasone. GC treatment was stable for at least 6 months at the time of 24-hour urine collection. Box-and-whisker plots represent median, interquartile range (box), and 5th and 95th percentiles (whiskers). Analyses were undertaken using the Kruskal-Wallis test with post hoc Dunn. *P ≤ 0.05, ***P ≤ 0.001. PT, pregnanetriol; THS, tetrahydro-11-deoxycortisol.

The analysis of the total 24-hour urine excretion revealed a significant reduction in the combined excretion of the markers of impaired 21-hydroxylase activity, the sum of 17OHP metabolites 17HP, pregnanetriol, and the 21-deoxycortisol metabolite PTONE, both after 4 days and 6 months of Chronocort treatment (all *P* < 0.05) (Fig. 4),] with lower excretion amounts than observed in patients treated with any other GC preparation (Fig. 5). Total classic pathway androgen metabolite excretion, An + Et, and excretion of the alternative androgen pathway metabolite 3*α*5*α*-17HP significantly decreased after Chronocort treatment to lower levels than observed with any other GC preparation (Figs. 4 and 5). The excretion of 11*β*-OH-An also appeared to decrease, albeit not significantly (Fig. 4). Attenuation of 11-oxygenated 19-carbon androgen synthesis in Chronocort-treated patients was at least similar to dexamethasone or prednisolone treatment and superior to the effects of hydrocortisone treatment (Fig. 5). Of note, 24-hour urinary excretion of 11-hydroxy-etiocholanolone and 11-oxo-etiocholanolone, which are exclusive GC metabolites (15) and therefore reflective of the amount of exogenous cortisol, showed a higher excretion in patients treated with conventional hydrocortisone than in patients taking Chronocort.

The effect of 6 months of Chronocort therapy on the diurnal rhythm of urinary steroid excretion in patients with CAH is shown in Supplemental Fig. 3. There was less variability seen across the three 8-hour periods in the excretion of the metabolites of CYP21A2 following Chronocort than prior to its initiation. Notably, the early morning surge (nighttime period, 23:00–7:00) in the activation of classic and alternative androgen pathway synthesis appeared diminished following Chronocort therapy [Supplemental Fig. 3(C)–3(F)].

## Discussion

In this study, employing 24-hour urinary steroid metabolome profiling, we could show that alternative pathway androgen synthesis contributes significantly to androgen excess in patients with CAH receiving chronic GC therapy, both via the 11-oxygenated 19-carbon androgen pathway and via DHT synthesis from 17OHP. In addition, we have identified the differential impact of conventional GC therapies and treatment with modified-release hydrocortisone (Chronocort) on steroid excretion in CAH, including their effects on alternative pathway androgen synthesis—namely, the alternative “backdoor” pathway to DHT and the 11-oxygenated C19 steroid pathway.

Elements of the alternative “backdoor” pathway to DHT were first described by Wilson *et al.* (5), reporting the synthesis of 5*α*-androstanediol from 17OHP, with 3*α*,5*α*-17HP as the intermediate in the fetal testis of the tammar wallaby pouch young. They hypothesized that this pathway could extend to the conversion of 5*α*-androstanediol to DHT, thereby achieving active androgen synthesis without the classic pathway intermediates DHEA, androstenedione, and testosterone. This led Auchus (3) to coin the term *backdoor pathway* for this alternative pathway to DHT synthesis. They later showed that the final step to DHT can indeed take place in the gonads of the brushtail possum, the tammar wallaby, and the short-tail opossum (16–18). Arlt *et al.* (4) were the first to suggest the relevance of the alternative pathway to DHT in humans, as an explanation for the virilization of newborn girls affected by CAH, using the example of CAH due to P450 oxidoreductase deficiency, which results in disruption of the classic androgen pathway. Although that work focused on the role of the alternative pathway in prenatal life, they postulated that synthesis of DHT via the alternative pathway is likely to occur or increase, respectively, if there is an increase in either the availability of its substrate 17OHP or the activity of 5*α*-reductase type 1 activity, which catalyzes the first step of the alternative pathway. Both progesterone and 17OHP are efficient substrates for the 5*α*-reductase activity of SRD5A1 (19), and both these steroids accumulate in CAH with impaired 21-hydroxylase activity. Homma *et al.* (20) showed increased urinary excretion of the alternative pathway intermediate 3*α*,5*α*-17HP in patients with CAH due to P450 oxidoreductase deficiency; P450 oxidoreductase serves as the electron donor enzyme to 21-hydroxylase, and therefore its disruption results in impaired 21-hydroxylase activity. Subsequently, Kamrath *et al.* (6) demonstrated increased 3*α*,5*α*-17HP in newly diagnosed and hence untreated patients with CAH due to 21OHD aged 1 day to 25 years, noting the highest excretion amounts in the neonatal period. In this study investigating the steroid metabolome in adult patients with CAH taking established GC therapy, we found that although classic androgen pathway activity was significantly reduced, there was significantly increased excretion of 3*α*,5*α*-17HP, indicating an increased relative contribution of alternative androgen pathway DHT synthesis to androgen excess in CAH also in adulthood and in patients receiving regular GC treatment.

We also found increased excretion of 11*β*-OH-An, the major metabolite of 11-oxygenated 19-carbon androgens. Of note, Kamrath *et al.* (6) also showed significantly increased excretion of 11*β*-OH-An in untreated patients with CAH. However, at the time, they considered 11*β*-OH-An a classic pathway metabolite, but in fact this steroid represents the major metabolite of 11*β*-hydroxy-androstenedione and other 11-oxygenated 19-carbon androgens (15), effectively the second alternative pathway to the synthesis of active androgens. Its end products, 11-keto-testosterone and 11-keto-DHT, have shown similar androgenic activity to testosterone and DHT (7–10). In a very recent publication, serum metabolome profiling by tandem mass spectrometry demonstrated 3- to 4-fold increased circulating concentrations of 11*β*-hydroxy-androstenedione, 11-keto-androstenedione, 11*β*-hydroxy-testosterone, and 11-keto-testosterone in patients with 21OHD (21). However, this was done in a cross-sectional cohort of patients with CAH with no detailed data on GC therapy available.

In our study, conventional GC therapy appeared to control the activity of the alternative androgen synthesis pathways less efficiently than classic pathway synthesis. The latter we even found to be significantly suppressed in patients with CAH, below the levels observed in healthy sex- and age-matched controls, indicative of relative GC overtreatment that is frequently observed in adult patients with CAH (22). Studying the diurnal variation of steroid excretion in our patients, we observed that the increased excretion of the alternative pathway metabolites 3*α*,5*α*-17HP and 11*β*-OH-An is most likely consequent to the early morning surge in ACTH, which is unopposed in patients with CAH taking conventional GC therapy.

By contrast, we found that Chronocort, a modified-release hydrocortisone preparation, exerted much improved control of alternative pathway-mediated androgen excess. Chronocort has been shown to yield cortisol delivery mimicking physiological cortisol secretion (13), resulting in significant normalization of circulating 17OHP and androstenedione levels in a previously published phase 2 study in patients with CAH (14). This effect was almost more impressively visible when studying the urines of this cohort of 16 patients in our study, with close to normalization of 17OHP metabolite excretion in Chronocort-treated patients. Conventional GC treatment never normalizes 17OHP secretion, and if present, this would be considered an indicator of significant over-replacement. However, near-normal diurnal provision of cortisol by Chronocort exerted superior control of 17OHP secretion and thereby also of both alternative androgen pathways driving androgen excess in CAH, which are both fed by the conversion of 17OHP, either to 11-oxygenated 19-carbons steroids or to 3*α*,5*α*-17HP and further downstream to DHT via the “backdoor pathway.” An alternative modified-release formulation of hydrocortisone, Plenadren, has immediate- and delayed-release actions but is licensed for use in adrenal insufficiency, where it is taken first thing in the morning as a once-daily medication (23), and was not studied here. In the only study to our knowledge to report the use of Plenadren in CAH, 6 patients with CAH were included in an open-label trial of Plenadren where body mass index, hemoglobin A1c, and quality of life were measured but androgens were not reported (24).

Importantly, in our study, the analysis of the exclusive cortisol metabolites 11*β*-hydroxy-etiocholanolone and 11-oxo-etiocholanolone (15) clearly indicated a higher excretion in the patients on conventional hydrocortisone treatment than in those treated with Chronocort, the modified-release hydrocortisone preparation. This means that the absolute amount of bioavailable cortisol was actually lower in Chronocort-treated patients with CAH, supporting the assumption that it was not the total amount of GC but the improved diurnal delivery of cortisol by Chronocort, and therefore the better control of the early morning ACTH and steroid surge, that results in the superior control of excess 17OHP and androgen production.

A limitation of our study was the fact the CAH patient groups receiving the 3 conventional GC preparations (hydrocortisone, prednisolone, and dexamethasone) were not matched for biochemical control at baseline and were studied cross-sectionally and not during a controlled crossover study. However, they were a cohort of considerable size recruited from 2 large specialist centers, which ensures a relative homogenization of clinical presentation. An advantage of our study was the inclusion of adult patients only, which allowed us to dissect androgen production in detail.

In conclusion, we have identified significant alternative androgen synthesis pathway activity in adult patients with CAH taking conventional GC therapy that persists despite suppression of classic pathway androgen production by relative GC overtreatment. However, we found that the modified-release hydrocortisone preparation Chronocort results in superior control of alternative pathway androgen production, most likely by reducing the early morning surge in excess 17OHP, which in CAH represents the major substrate for both the alternative androgen pathway to DHT and the 11-oxygenated androgen pathway.
