# Supplementary material for: Modified-Release and Conventional Glucocorticoids and Diurnal Androgen Excretion in Congenital Adrenal Hyperplasia
Source: J Clin Endocrinol Metab. 2016 Nov 15;102(6):1797–806. doi: 10.1210/jc.2016-2855 (PMC5470768; doi:10.1210/jc.2016-2855)
Supplement: Supplementary file 1 [file jc.2016-2855.s1.docx]

**Suppl. Table 1:** **Summary of urinary steroid metabolites and their origin and significance.**

| **Abbreviation** | **Common name** | **Chemical name** | **Metabolite of** |
| --- | --- | --- | --- |
| 17HP | 17α-hydroxypregnanolone | 5β-pregnane-3α,17α-diol-20-one | 17α-hydroxyprogesterone (substrate of 21-hydroxylase) |
| PT | Pregnanetriol | 5β-pregnane-3α,17α,20α-triol | 17α-hydroxyprogesterone (substrate of 21-hydroxylase) |
| PTONE | Pregnanetriolone | 5β-pregnane-3α,17α,20α-triol-11-one | 21-deoxycortisol (only generated from 17OHP by CYP11B1 in the absence of appreciable 21-hydroxylase activity) |
| THS | Tetrahydro-11-deoxycortisol | 5β-pregnane-3α,17α,21-triol-20-one | 11-deoxycortisol (product of 21-hydroxylase) |
| An | Androsterone | 5α-androstan-3α-ol-17-one | 5α-reduced androgen metabolites derived from steroids within the classic androgen pathway (androstenedione, testosterone) and 5α-dihydrotestosterone (DHT) generated via classic and alternative pathway |
| Et | Etiocholanolone | 5β-androstan-3α-ol-17-one | 5β-reduced androgen metabolites derived from steroids within the classic androgen pathway (androstenedione, testosterone) |
| 3α5α17HP | 5α-17-hydroxy-pregnanolone | 5α-pregnane-3α,17α-diol-20-one | 17α-hydroxyprogesterone (5α-17-hydroxy-pregnanolone is the intermediate and signature metabolite of the alternative pathway to DHT synthesis) |
| 3α5α17HP/An |  |  | Ratio representative of the contribution of the alternative pathway to DHT synthesis. |
| 11β-OH-An | 11β-hydroxyandrosterone | 5α-androstan-3α,11β-diol-17-one | Major metabolite of 11-oxygenated 19-carbon androgens including and downstream of 11β-hydroxyandrostenedione |

**Suppl. Figure Legends**

**Suppl. Fig. 1:** **Effect of conventional glucocorticoid treatment on 24-hour urinary steroid metabolite excretion in 55 patients with CAH due to 21OHD**.

Data are presented for patients managed with hydrocortisone (n=13; dark grey), prednisolone (n=27; white) or dexamethasone (n=15; light grey). Urinary excretion of 3α5α17HP available for 38 of the total CAH cohort; 11 hydrocortisone, 21 prednisolone and 6 dexamethasone. Box-and-whisker plots represent median, interquartile range (box) and 5^th^ and 95^th^ percentiles (whiskers). Analyses were undertaken using the Kruskal-Wallis test with post-hoc Dunn. *, p≤0.05. ***, p≤0.001.

**Suppl. Fig. 2: Effect of Chronocort treatment delivered on diurnal urinary steroid excretion in 16 patients with CAH.**

Results are shown for timed urine collections taken at initiation of therapy (baseline: BL) and after six months of treatment (M6) and are depicted for the following three time periods: night (23:00-07:00; dark grey), morning (07:00-15:00; white) and evening (15:00-23:00; light grey). Data are shown as µg/8 hours and are presented as box-and-whisker plots to represent median, interquartile range (box) and 5^th^ and 95^th^ percentiles (whiskers). Data were analyzed using the Friedman test; none of the comparisons reached significance.

.

**Supp. Fig. 3:** **Sex-specific analysis of** **24-h urinary steroid excretion in 55 CAH patients and 60 healthy sex- and age-matched controls.** Data are shown for male (M) and female (F) patients as µg/24 hours and presented as box-and-whisker plots to represent median, interquartile range (box), and 5^th^ and 95^th^ percentiles (whiskers). Analyses were undertaken using the Kruskal-Wallis test with post-hoc Dunn. * p≤0.05. *** p≤0.001 as indicated for the comparison CAH vs. controls and males vs. females.

**Suppl. Fig. 1:** **Effect of conventional glucocorticoid treatment on 24-hour urinary steroid metabolite excretion in 55 patients with CAH due to 21OHD**.

**
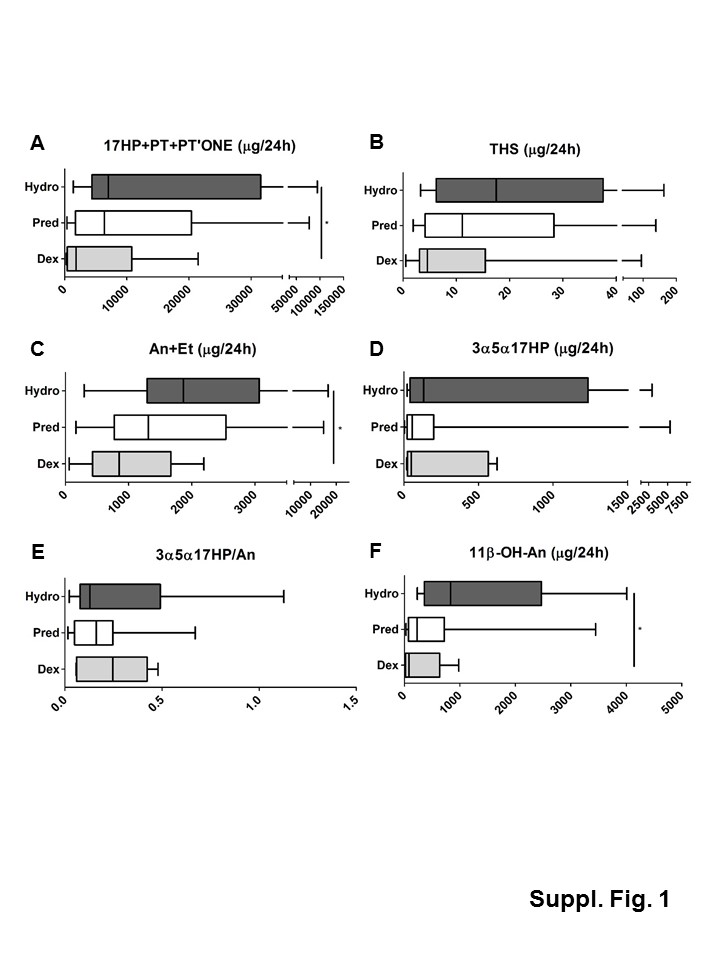
**

**Suppl. Fig. 2: Effect of Chronocort treatment delivered on diurnal urinary steroid excretion in 16 patients with CAH.**

**
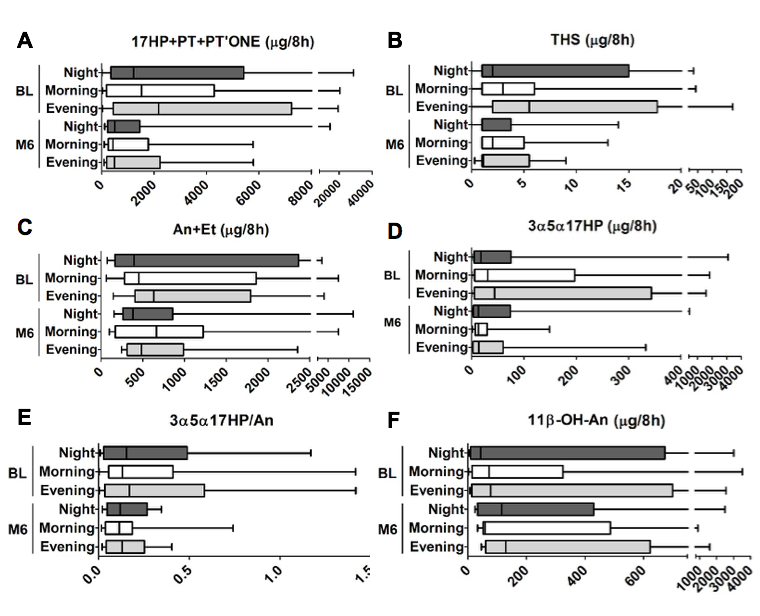
**

**Supp. Fig. 3:** **Sex-specific analysis of 24-h urinary steroid excretion in 55 CAH patients and 60 healthy sex- and age-matched controls.**

**
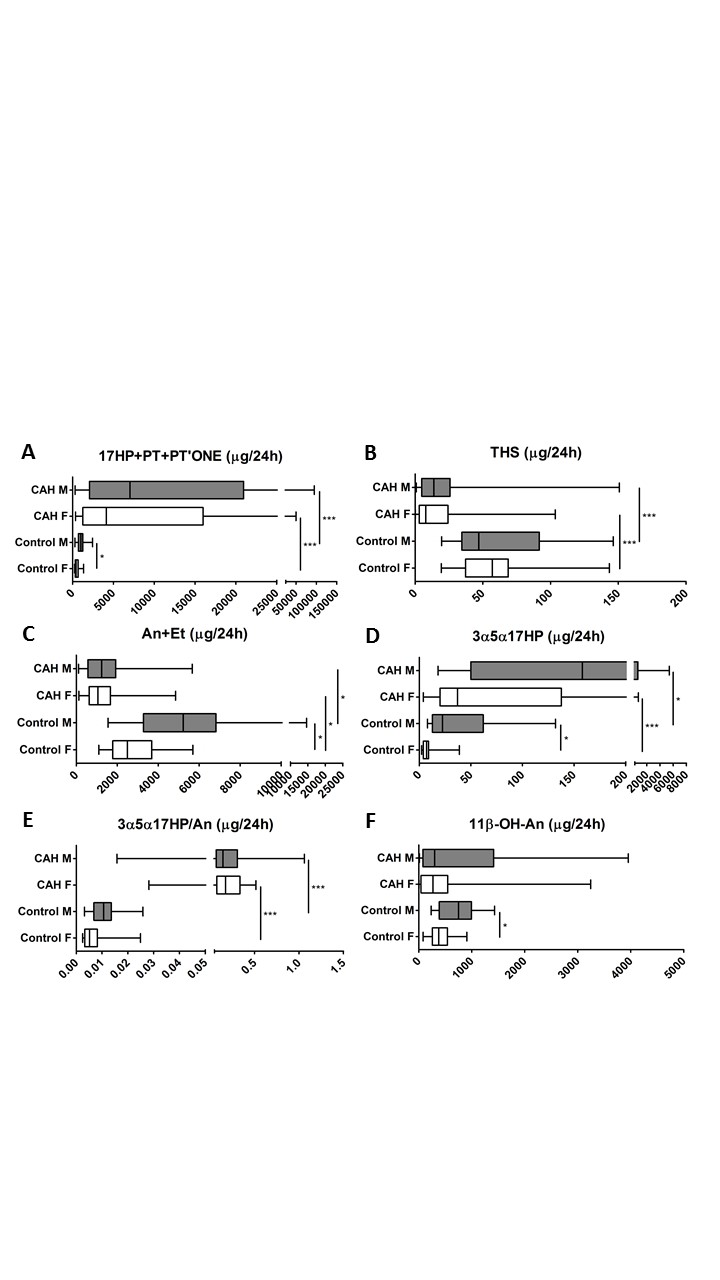
**
